# A random forest approach to the detection of epistatic interactions in case-control studies

**DOI:** 10.1186/1471-2105-10-S1-S65

**Published:** 2009-01-30

**Authors:** Rui Jiang, Wanwan Tang, Xuebing Wu, Wenhui Fu

**Affiliations:** 1 MOE Key Laboratory of Bioinformatics and Bioinformatics Division, TNLIST/Department of Automation, Tsinghua University, Beijing 100084, PR China

## Abstract

**Background:**

The key roles of epistatic interactions between multiple genetic variants in the pathogenesis of complex diseases notwithstanding, the detection of such interactions remains a great challenge in genome-wide association studies. Although some existing multi-locus approaches have shown their successes in small-scale case-control data, the "combination explosion" course prohibits their applications to genome-wide analysis. It is therefore indispensable to develop new methods that are able to reduce the search space for epistatic interactions from an astronomic number of all possible combinations of genetic variants to a manageable set of candidates.

**Results:**

We studied case-control data from the viewpoint of binary classification. More precisely, we treated single nucleotide polymorphism (SNP) markers as categorical features and adopted the random forest to discriminate cases against controls. On the basis of the gini importance given by the random forest, we designed a sliding window sequential forward feature selection (SWSFS) algorithm to select a small set of candidate SNPs that could minimize the classification error and then statistically tested up to three-way interactions of the candidates. We compared this approach with three existing methods on three simulated disease models and showed that our approach is comparable to, sometimes more powerful than, the other methods. We applied our approach to a genome-wide case-control dataset for Age-related Macular Degeneration (AMD) and successfully identified two SNPs that were reported to be associated with this disease.

**Conclusion:**

Besides existing pure statistical approaches, we demonstrated the feasibility of incorporating machine learning methods into genome-wide case-control studies. The gini importance offers yet another measure for the associations between SNPs and complex diseases, thereby complementing existing statistical measures to facilitate the identification of epistatic interactions and the understanding of epistasis in the pathogenesis of complex diseases.

## Background

Recent advances in human and medical genetics have made it widely accepted that Mendelian disorders that are caused by individual genetic variants are rare, whereas complex diseases that are speculated to be caused by multiple genetic variants, their interactive effects, and/or their interactions with environment factors are common [1,2]. The interactive effect between two or more genetic variants is typically referred to as *epistasis*, which is supposed to contribute to complex diseases ubiquitously via the sophisticated regulatory mechanisms in the molecular level of human genetics [3]. Biomedical studies have also been confirming the existence of epistasis in many complex diseases, including myocardial infarction [4], breast cancer [5], hypertension [6], atrial fibrillation [7], diabetes mellitus type 2 [8], AIDS [9], and many others. The detection of epistatic interactions therefore plays a key role in the understanding of the pathogenesis of complex diseases.

Nevertheless, most statistical approaches that have demonstrated remarkable successes in the detection of genetic variants underlying Mendelian diseases have impaired explanatory power in the identification of epistatic interactions responsible for complex diseases [10]. For example, family-based linkage analysis that uses a transmission model to explain the pattern of inheritance of phenotypes and genotypes exhibited in a pedigree works well for Mendelian diseases, but it is ineffective when a single locus fails to explain a significant fraction of a disease [1,2].

On the other hand, with the completion of human genome project, new opportunities and challenges have been presented for uncovering the genetic basis of complex diseases via genome-wide association studies [3,11]. With the accumulation of well-phenotyped cases and carefully selected controls, as well as the emergence of high-throughput genotyping techniques, it becomes urgent to develop effective methods for the detection of epistatic interactions.

To embrace the opportunities, a number of multi-locus approaches have been developed. For example, Hoh *et al *proposed a trimming, weighting, and grouping approach that used the summation of statistics on the basis of single-locus marginal effects and the Hardy-Weinberg equilibrium (HWE) for hypothesis testing [12]. Nelson *et al *put forward a combinatorial partitioning method (CPM) that exhaustively searched for the combinatorial genotype group that has the most significant difference in the mean of the responding continuous phenotype [13]. Culverhouse *et al *modified CPM by ignoring partitions that combined individual genotypes with very different mean trait values [14]. Millstein *et al *developed a pre-screening strategy to reduce the number of tests with the use of a focused interaction testing framework (FITF) [15]. Chatterjee *et al *used Turkey's 1-degree-of-freedom model to detect interacting loci from different region [16]. Ritchie *et al *adopted an exhaustive search strategy to detect combinations of loci with the highest classification capability and named their method multifactor-dimensionality reduction MDR [5]. Zhang and Liu introduced a Bayesian epistasis association mapping (BEAM) method that integrated a Bayesian model with a Metropolis-Hasting algorithm to infer the probability that each locus was associated with the susceptibility of a disease [17]. They also proposed the use of a new *B *statistic instead of the standard *χ*^2 ^statistic. Many machine learning methods have also been applied to this research field from the viewpoint of binary classification and feature selection [18-24].

The effectiveness of most of these methods for genome-wide case-control data has not yet been validated. Besides, many methods rely heavily on the exhaustive search for combinations of multiple loci. This strategy, though feasible when the number of candidate SNPs is small, can hardly be computationally practical for genome-wide association studies in which the number of candidate SNPs is typically very huge. For example, a study on Age-related Macular Degeneration (AMD) has genotyped more than 100 thousand single nucleotide polymorphism (SNP) markers for 96 patients and 50 unaffected people [25]. It has also become very common to genotype 400~500 thousand SNP markers for hundreds of cases and controls in recent genome-wide association studies [26,27]. With such dense SNPs being genotyped, methods based on the exhaustive search are computationally impractical due to the vast number of combinations of SNPs. One of the main challenges for genome-wide association studies is therefore to design computational methods that are able to reduce the search space for epistatic interactions from an astronomic number of all possible combinations of SNPs to a manageable set of candidates.

In this paper, we study case-control data from the viewpoint of binary classification. Specifically, we treat cases as positive samples and controls as negative samples, and we use SNP markers as categorical features that have three possible values (i.e., the three genotype values at a locus). With this notion, we adopt the *random forest *[28] that has been widely used in bioinformatics fields such as the selection of genes [19,20], the identification of gene-gene interactions [19,22], and the detection of causative nonsynonymous SNPs [29,30] as the classifier to discriminate cases against controls, with an emphasis on the contribution of each SNP to the classification performance. For this purpose, we first run a random forest with all SNPs to obtain the *gini importance *of each SNP and then use a sliding window sequential forward feature selection (SWSFS) algorithm to select a subset of SNPs that can minimize the classification error. Since this subset typically contains only a small number of SNPs (e.g., ~100), we are able to enumerate all possible *k*-way (*k *= 1, 2, 3) interactions of the candidate SNPs and test for statistical significance their associations with the disease risk.

The above approach, named *epi*Forest (detection of *epi*static interactions using random Forest), was compared with three existing methods (BEAM [17], the stepwise logistic regression [11], and the classical *χ*^2 ^test) on three simulated disease models [11]. The results showed that *epi*Forest was comparable to, sometimes more powerful than, these methods. We further applied the proposed approach to a genome-wide case-control dataset for AMD that contains 116,204 SNPs genotyped for 96 cases and 50 controls [25] and selected a subset of 84 SNPs that can minimize the classification error. Further statistical tests successfully detected from these candidates two SNPs (rs380390 and rs1329428) that were reported to be associated with this disease.

## Results

### Principles of *epi*Forest

The classical approach to the detection of single-locus association fits a full logistic regression model with a parameter for each observed genotype and then tests the significance of the fitted model via a *χ*^2 ^approximation of the likelihood ratio test statistic [11]. Alternatively, a *χ*^2 ^test with up to 2 degrees of freedom can be directly applied to the contingency table that records the number of cases and controls for each genotype to test whether the distributions of SNPs for the case and control populations are significantly different. To ensure the overall type I error not exceeding a preset significance level *α*, a Bonferroni correction is typically applied by multiplying the *p*-values with the number of SNP markers *L *(or equivalently setting the significance level to *α*/*L*).

Similarly, in order to detect the epistatic interaction of two loci, a full logistic regression model with at most 9 parameters can be fitted and tested, and the *p*-values should be multiplied by *L*(*L*-1)/2 according to the Bonferroni correction [11]. Because the number of SNPs is typically huge (e.g., several hundred thousand) in genome-wide case-control studies, an exhaustive search for all possible combinations of SNPs is computationally impractical. To overcome this limitation, the stepwise logistic regression approach first selects a small fraction (*ε*, e.g., 10%) of loci according to the significance of their single-locus associations and then tests the interactions between the selected loci [11]. The determination of the fraction *ε *is, however, not guided. An approach that is able to automatically determine such a small set of good candidate markers is therefore preferred.

**Figure 1:**
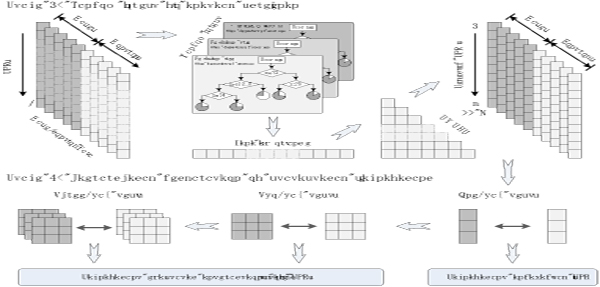
**Principles of *epi *Forest**. In the first stage, a random forest is trained with all SNPs to obtain the gini importance of each SNP, and a sliding window sequential forward feature selection (SWSFS) algorithm is used to select a subset of candidate SNPs that can minimize the classification error. In the second stage, statistical tests on the basis of the B statistics are applied to detect significant one-, two-, and three-way epistatic interactions.

For this purpose, we propose to use *epi*Forest, a two-stage approach as illustrated in Figure 1, for the detection of epistatic interactions. A case-control study can be thought of as a binary classification problem, in which we treat cases as positive samples and controls as negative samples, and we target on discriminating cases against controls. The SNP markers can be used as categorical features with three possible values in this classification formulation.

With this notion, in the first stage, we use an ensemble learning technique called random forest [28] with all SNPs to do the classification, while the objective is to obtain the contribution, measured by *gini importance*, of each SNP (see Methods). Then, a *Sliding Window Sequential Forward feature Selection *(SWSFS) algorithm that adds one SNP at a time from the most significant SNP to the least significant one is applied to greedily search for a small subset of SNPs that could minimize the classification error (see Methods). After this step, a small set of *l *(<<*L*, the total number of SNP markers) candidate SNPs that have the most significant contribution to the discrimination of cases against controls is selected.

In the second stage, a hierarchical procedure is adopted to declare the statistical significance that the candidate SNPs are associated with the disease risk. Let *α *be a preset significance level (e.g., 0.05). In the one-way tests, we apply a statistical test with the use of the *B statistic *proposed by Zhang and Liu (see [17] and Methods) to every candidate SNP and report all SNPs whose *p*-values are less than *α *after Bonferroni corrections for *L *tests. In the two-way tests, we apply the *B *statistic to all two-way interactions of the candidates, and report interactions whose *p*-values are less than *α *after Bonferroni corrections for *L*(*L*-1)/2 tests. In this procedure, if both SNPs in an interaction have already been reported in the one-way tests, we skip the test for their interaction; if one of the SNPs has already been reported in the one-way tests, we use a conditional *B *statistic for testing the interaction; if neither SNPs in an interaction has been reported in the one-way tests, we use the B statistic for testing the interaction. Similarly, in the three-way tests, we apply the *B *or conditional *B *statistics to all three-way interactions of the candidates, and report those with *p*-values less than *α *after Bonferroni corrections for *L*(*L*-1) (*L*-2)/6 tests.

### Performance of *epi*Forest

In order to demonstrate the performance of *epi*Forest, we compared it with three existing methods, BEAM [17], the stepwise logistic regression [11], and the standard single-locus *χ*^2 ^test, on three simulated disease models.

BEAM uses a Bayesian model with the Metropolis-Hasting algorithm to partition SNP markers into three groups: a group *G*_0 _containing markers unlinked to the disease, a group *G*_1 _including markers contributing independently to the disease, and a group *G*_2 _that is composed of markers jointly influencing the disease. After the partition step, candidate SNPs are further tested for significance with the use of the *B *statistic [17]. In BEAM, there are two prior probabilities need to be pre-determined: the probability that each marker belongs to *G*_1 _and that of *G*_2_. In our studies, we set both priors to 0.001. The stepwise logistic regression first selects the most significant *ε *fraction of SNPs on the basis of their marginal effects, and then tests all two-way interactions of these SNPs using logistic regressions with likelihood ratio tests [11]. We use *ε *= 10% in our studies and further test all three-way interactions of the candidates besides the two-way interactions. The classical single-locus *χ*^2 ^test (with at most 2 degrees of freedom) is used as a benchmark in this comparison.

We considered three disease models with different characteristics (see [11] and [17] for details). Briefly, model 1 contains two disease loci that contribute to the disease risk independently. Model 2 is similar to model 1, except that the disease risk is present only when both loci have at least one disease allele. Model 3 is a threshold model in which additional disease alleles at each locus do not further increase the disease risk. Assuming the disease prevalence to be 0.1 for all disease models, each model has three parameters associated: the marginal effect of each disease locus (*λ*), the minor allele frequencies (MAF) of both disease loci, and the strength of linkage disequilibrium (LD) between the unobserved disease locus and a genotyped locus (*r*^2^) [31]. To enumerate all possible combinations of these parameters is impossible. We therefore selected only some typical values for each parameter. For *λ*, we set it to 0.3, 0.5, and 1.0 for model 1, 2, and 3, respectively. For MAF, we considered four values, 0.05, 0.1, 0.2, and 0.5, for each model. For *r*^2^, we simulated for each model two values, 0.7 and 1.0. There were therefore 8 parameter settings for each disease model and a total of 24 comparisons in our simulation studies.

For each parameter setting of each model, we simulated 100 datasets, each of which contains 1,000 markers genotyped for 1,000 cases and 1,000 controls. The minor allele frequency for each non-disease marker is randomly chosen from a uniform [0.0. 0.5] distribution. The performance of a method on a specific parameter setting is measured by the power, defined as the fraction of simulated datasets in which all disease loci are identified at the significance level *α *= 0.05 after the Bonferroni correction.

**Figure 2:**
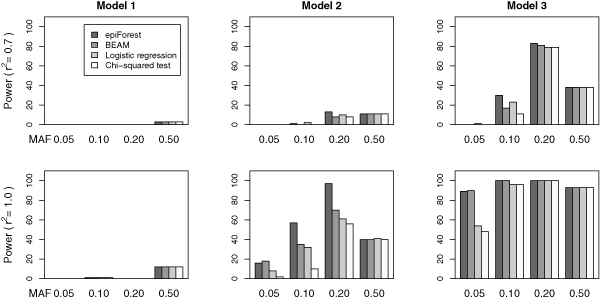
**Performance of *epi *Forest**. The power of *epi*Forest is compared with that of BEAM [17], the stepwise logistic regression [11], and the *χ*^2 ^test on 24 parameter settings of 3 disease models. 100 datasets, each containing 1,000 markers for 1,000 cases and 1,000 controls, are simulated for each parameter setting. The power is defined as the fraction of datasets in which all disease loci are identified at the significance level 0.05 after the Bonferroni correction.

The simulation results are shown in Figure 2. An overall impression is that the power of *epi*Forest is comparable to, sometimes higher than, that of BEAM and the stepwise logistic regression, while all these three methods are superior to the *χ*^2 ^tests. Specifically, all methods achieve similar performance in model 1, regardless of the LD strength. The reason behind this observation is that model 1 is actually a non-epistasis model, in the sense that the two causative loci contribute to the disease risk independently. Therefore, all methods for epistasis detection achieve similar performance as the single-locus *χ*^2 ^test, suggesting that the more complex models have little effects in this situation. In model 2, *epi*Forest and BEAM show their superior performance when the minor allele frequencies of the disease markers are small. This might be attributed to the benefit of using the more powerful *B *statistic. We also notice that the standard *χ*^2 ^test, as a single-locus search method, performs poorly when the minor allele frequencies of the disease markers are small, suggesting the necessity of developing multi-locus approaches in the search for markers that have epistatic interactions. In model 3, we have similar observations as in model 2.

### Effectiveness of the SWSFS algorithm

The subset of candidate markers that are likely to be associated with the disease risk is screened out with the use of a sliding window sequential forward feature selection (SWSFS) algorithm, given the gini importance provided by the initial run of the random forest (see Methods). It is therefore necessary to see how many markers are typically selected by this algorithm.

**Figure 3:**
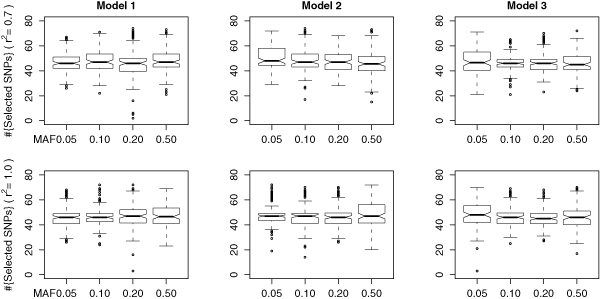
**Number of SNP markers selected by *epi *Forest**. The median of the number of markers selected by the sliding window sequential forward feature selection (SWSFS) algorithm is around 45, while the maximum is in general less than 80, suggesting the capacity of the SWSFS algorithm.

For each parameter setting of the disease model, we plot the number of markers selected by the SWSFS algorithm in Figure 3. From these box plots, we can see that the median of the number of selected markers is around 45 for every parameter setting, and the upper bound of this number is generally less than 80. In other words, the SWSFS algorithm is capable of shrinking the search space from 1,000 SNPs to, typically, about 45 markers, thereby facilitating further statistical tests for epistatic interactions within this small set of candidates.

In our simulation studies, the power of *epi*Forest is in general superior than that of the stepwise logistic regression, while the parameter *ε *(the fraction of candidate markers screened on the basis of their marginal effects) in the stepwise logistic regression is set to 10% (100 markers), which is generally greater than the number of candidates suggested by the SWSFS algorithm. These facts suggest that *epi*Forest can more precisely pinpoint the candidate SNPs that might be associated with the disease risk, and this procedure is fully automated.

The observations from Figures 2 and 3 suggest the feasibility of using machine learning methods to select a set of candidate markers that are likely to be associated with the disease risk, thereby reducing the search space for epistatic interactions from a large number of SNPs to a small number of selected candidates. Traditional approaches such as the stepwise logistic regression uses the marginal importance of individual markers as the criterion to select the subset of candidate for further exploration, and the size of the subset remains as a free parameter whose determination is not guided. With the use of *epi*Forest, however, the subset is automatically determined as the one that can minimize the classification error, therefore providing an automated initial screening. On the other hand, because the criterion used by *epi*Forest (gini importance) is intrinsically different from the *p*-value provided by likelihood ratio tests that is used in the stepwise logistic regression, it is possible that the gini importance can complement statistical criteria to achieve a better search for epistatic interactions. The results also demonstrate the power of the *B *statistic over the likelihood ratio test statistic and the *χ*^2 ^statistic, because both *epi*Forest and BEAM are in general more powerful than the stepwise logistic regression and the *χ*^2 ^test.

### Application to AMD

In simulation studies on 1,000 SNPs, *epi*Forest is comparable to, sometimes more powerful than, three existing methods. Nevertheless, studies have shown that a number of 30,000 to 500,000 common SNPs may be required for genotyping in real genome-wide case-control studies [32,33]. It is therefore necessary to show whether *epi*Forest is able to handle such large data in real genome-wide association studies.

For this purpose, we applied *epi*Forest to an Age-related Macular Degeneration (AMD) dataset [25], which contained 116,204 SNPs genotyped with 96 cases and 50 controls. As suggested in [25], we removed nonpolymorphic SNPs and those that significantly deviated from Hardy-Weinberg Equilibrium (HWE), and we removed all SNPs that had no reference SNP ID or had more than 5 missing genotypes to ensure the high quality of the remaining data. After the filtering, there remained 95,986 SNPs.

**Figure 4:**
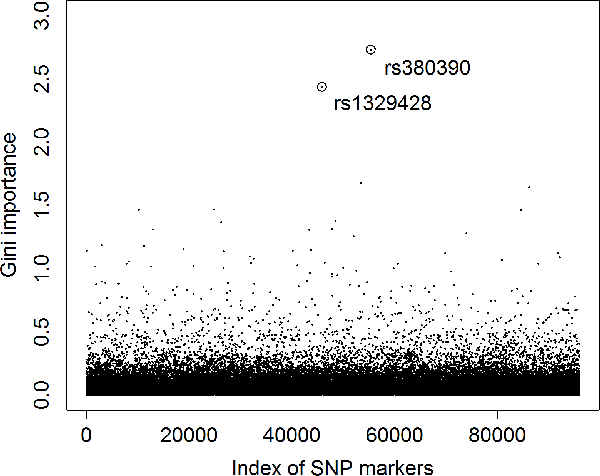
**Gini importance of the SNPs in the AMD dataset**. *x*-axis is the index of SNP markers. *y*-axis is the gini importance. The two circles represent the two SNPs that are already identified in literature [25].

We first run a random forest with the use of all SNPs as categorical features to discriminate the 96 cases against the 50 controls. The aim was to obtain the gini importance, indicating the contribution to the classification accuracy, of each SNP. We used one million trees in the construction of the random forest and repeated the experiment ten times to reduce random effects. The gini importance for each SNP was averaged over the resulting ten forests and shown in Figure 4.

It is interesting to see from this figure that the two SNPs, rs380390 and rs1329428, that were reported to be associated with AMD in literature [25], have the largest gini importance among all SNPs. Specifically, the gini importance is 2.73 for rs380390 and 2.44 for rs1329428, while the Bonferroni-corrected *p*-values are 0.0043 and 0.14 for rs380390 and rs1329428, respectively, according to *χ*^2 ^tests under the assumption of Hardy-Weinberg equilibrium [25]. These observations suggest that higher gini importance may indicate lower *p*-value.

**Figure 5:**
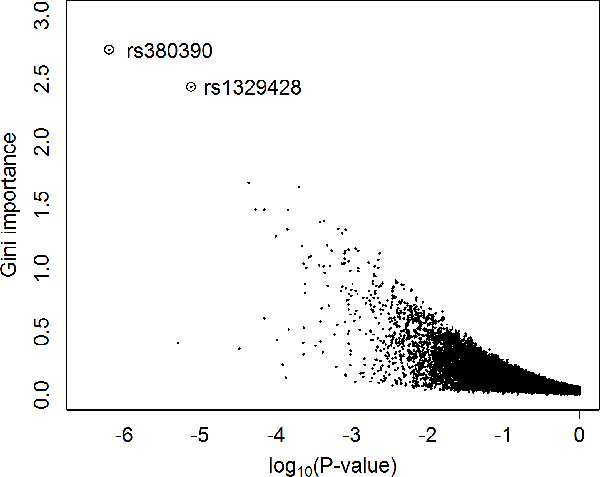
**Relationship between the gini importance and the p-value for the B statistic**. *x*-axis is the logarithm (base 10) of the p-value for the B statistic. *y*-axis is the gini importance.

We then plotted the relationship between the gini importance values and the *p*-values (without Bonferroni correction) for *B *statistics of all the 95,986 SNPs in Figure 5. We observed that, in general, larger gini importance values imply smaller *p*-values. In other words, the gini importance has a strong negative correlation with the *p*-value. In details, their Pearson's correlation coefficient (PCC) is -0.59, and is very significant with a *p*-value less than 2.2 × 10^-16 ^(given by R). This observation suggests that the gini importance from the viewpoint of machine learning may complement the *p*-value from the statistics point of view to offer yet another measure for the associations between SNPs and complex diseases.

**Figure 6:**
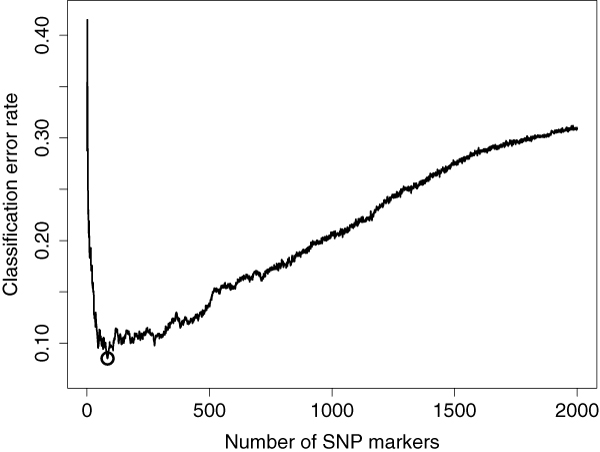
**Classification error rates of random forests using up to the first 2,000 most important SNPs**. *x*-axis is the number of SNP markers used. *y*-axis is the classification error rate given by the random forest (one million trees). The circle (with 84 markers and an error rate of 8.5%) represents the minimum in this curve.

To illustrate the sliding window sequential forward feature selection (SWSFS) algorithm, we plotted the classification error rate of random forests using up to the first 2,000 most important SNPs, as shown in Figure 6. The minimum classification error (8.5%) occurs when the first 84 most important SNPs (on the basis of their gini importance) are used. With the use of the SWSFS algorithm (window-size setting to 20, see Methods), only the first 104 random forests need to be constructed, therefore saving the computational expenses. It is worth noting that the minimal classification error, the number of the SNPs used, and the subset of SNPs are automatically determined by the SWSFS algorithm without the participation of human. When compared with the stepwise logistic regression method in which the fraction of candidate markers need to be manually determined for further investigation, our approach can provide an automated means of determining this critical value.

There has yet no report about interactions associated with AMD from this dataset thus far. The main reason might be the small sample size of 146 individuals is insufficient for detecting subtle epistatic interactions [17]. In our study, we also find no significant interactions after the Bonferroni correction. Nevertheless, we still report in Table 1 the top 5 two-way interactions and the top 5 three-way interactions that have the smallest *p*-values before the Bonferroni correction. It is interesting to see that neither rs6104678 nor rs7863587 is significant for single-locus association (actually there are 9 SNPs having smaller *p*-values then rs6104678 and 14 SNPs being more significant than rs7863587), whereas their combination has the smallest *p*-value among all two-way interactions. We can also see that rs1394608 appears in 3 out of 5 two-way interactions, and rs7104698 appears in all 5 three-way interactions. Certainly, these observations need to be further studied in depth with the use of more case-control samples, and functional tests are necessary to confirm whether these interactions have true associations with AMD. Although these studies are beyond the scope of this paper, we hope that, from Table 1, some clues could be provided for the exploration of epistatic interactions in this complex disease. Note that, some of these interactions have been previously detected independently by another genome-wide association study method that is developed by the authors to identify epistatic modules via the integration of Bayesian models and Markov Chain Monte Carlo strategies.

**Table 1:** Top 5 two-way and top 5 three-way interactions in AMD that have the smallest p-values (for the B statistics) before the Bonferroni correction.

SNP Interaction	*p*-value
(rs6104678, rs7863587)	1.28 × 10^-7^
(rs3743175, rs1394608)	3.06 × 10^-7^
(rs2828155, rs1394608)	3.06 × 10^-7^
(rs4292478, rs1394608)	7.29 × 10^-7^
(rs6104678, rs10512174)	7.68 × 10^-7^
(rs2347060, rs3758141, rs7104698)	5.57 × 10^-9^
(rs2347061, rs3758141, rs7104698)	5.57 × 10^-9^
(rs2347060, rs10503640, rs7104698)	6.91 × 10^-9^
(rs2347061, rs10503640, rs7104698)	6.91 × 10^-9^
(rs2347060, rs1557753, rs7104698)	1.07 × 10^-8^

## Discussion

The development of *epi*Forest is motivated by the following two facts: (1) most existing approaches use pure statistical methods and (2) in the stepwise logistic regression, the selection for the subset of candidate SNPs is not guided. Accordingly, the main contribution of our approach includes: (1) the incorporation of the random forest into case-control studies and (2) the automated screening of the candidate SNPs for further statistical analysis.

The random forest has several advantages over other classifiers in the studies for case-control data. First, and of the most interest, the random forest can natively provide the gini importance that measures the contribution of individual features (SNPs) to the classification. We have also shown that the correlation between this importance measure and the *p*-value for the *B *statistic is strongly negative. In this sense, the gini importance may complement the *p*-value to offer yet another useful measure for the associations between SNPs and complex diseases. Second, the random forest needs no extra cross-validation for evaluating the classification performance, thereby greatly reducing the computational time. Third, as a classical ensemble learning method, the procedures for constructing decision trees in the forest are mutually independent, hence very suitable for large scale parallel computation or hardware acceleration.

The *epi*Forest framework can also be extended from the following directions. First, the gini importance can itself serve as a statistic and be used with the permutation test to directly offer a *p*-value. Second, the random forest has an experimental means of estimating interactions between two features. For each tree, features can be ranked on the basis of their gini decreases, and the absolute difference in the ranks of every two features can be calculated. Averaging this difference over all trees, one obtains a measure for the interaction of every pair of features [17]. It is therefore interesting to analyze the relationship between this measure and statistical measures such as the *p*-value.

Certainly, our approach is not intended to take the place of existing statistical methods for detecting epistatic interactions. Instead, we are interested in showing how machine learning approaches can complement statistical methods to facilitate the exploration of interactions between multiple SNPs, because epistasis plays such an important role in the pathogenesis of complex diseases, and the detection of epistasis still remains a great challenge and needs to be studied from different perspectives.

## Conclusion

In this paper, we studied case-control data from the viewpoint of binary classification. We treated cases as positive samples and controls as negative samples, and used SNP markers as categorical features. We adopted random forest to discriminate cases against controls, while the focus was to obtain the gini importance to measure the contribution of each SNP to the classification performance. On the basis of this measure, a sliding window sequential forward feature selection (SWSFS) algorithm was proposed to automatically determine a subset of candidate SNPs that were most likely to be associated with the disease. A hierarchical procedure with the use of the *B *statistic was applied to declare statistical significance of up to three-way interactions within this set of candidates. This framework, including the random forest and the SWSFS algorithm for initial screening, and the hierarchical procedure for declaring statistical significance, was named *epi*Forest.

We compared the proposed approach with three existing methods, including BEAM [17], the stepwise logistic regression [11], and the *χ*^2 ^test, on three simulated disease models [11]. The results showed that the power of *epi*Forest was comparable to, sometimes higher than, that of the other methods.

We further applied *epi*Forest to a real genome-wide case-control dataset of AMD. The SWSFS algorithm automatically selected a set of 84 SNP markers. It was interesting to see that the two SNPs (rs380390 and rs1329428) already reported as linked to this disease [25] had the highest gini importance. A strong negative correlation between the gini importance and the *p*-value for the *B *statistic was also observed.

## Methods

### Random forest

The random forest is an ensemble learning methodology originated by Leo Breiman (see [28] for details). The basic idea of ensemble learning is to boost the performance of a number of weak learners via a voting scheme, where a weak learner can be an individual decision tree, a single perceptron/sigmoid function, or other simple and fast classifiers. As for the random forest, its hallmarks mainly include (1) bootstrap resampling, (2) random feature selection, (3) full depth decision tree growing, and (4) Out-of-bag (OOB) error estimate.

Given a set of *N *binary labelled training samples, where **x**_*i *_(*i *= 1, 2,..., *N*) is a vector of predictor variables (features) and *y*_*i *_the response variable (class label), a random forest targets on generating a number of *M *decision trees from these samples. For each tree, the same number of *N *samples is randomly selected with replacement (bootstrap resampling) to form a new training set, and the samples not selected are called out-of-bag (OOB) samples. Using this new training set, a decision tree is grown to the largest extent possible without any pruning according to the CART methodology [34], while in the split of each node, only a small number of *m *randomly selected features instead of all predictor variables is considered (random feature selection). Repeating the creation of a decision tree *M *times, we have a number of *M *distinct decision trees, forming a randomly generated "forest."

Unlike most machine learning methods that need to resort to cross-validation for the estimation of classification error, the random forest can natively estimate an out-of-bag (OOB) error in the process of constructing the forest, and this estimate is claimed to be unbiased in many tests [28]. With the construction of a decision tree, each OOB sample is tested, and its (OOB) classification result is collected. Upon the finish of constructing the entire forest, OOB classification results for each sample are used to determine a decision for this sample via a majority voting rule. The fraction of decisions that disagree with the true class label is then the OOB error estimate.

These characteristics make the random forest suitable for handling large-scale samples with thousands of features and thus gaining a wide spectrum of applications in bioinformatics such as the selection of genes [19,20], the identification of gene-gene interactions [19,22], and the detection of causative nonsynonymous SNPs [29,30]. In our studies, we use the "randomForest" package in R. The number of trees (*M*) and the number of features randomly selected in each node (*m*) are referred to as *ntree *and *mtry *(with the default value $\left\lfloor \sqrt{\#\{\text{SNPs}\}}\right\rfloor$) in this package, respectively. Detailed discussion about the effects of these parameters to the classification performance can be found in [20] and [28].

### Gini importance

Suppose that *η*, a node of a decision tree *T*, contains a number of *n *samples, in which *n*_0 _are negative and *n*_1 _= *n *- *n*_0 _are positive. The relative frequencies of the negative and positive samples, *f*_0 _and *f*_1_, respectively, can then be estimated as

*f*_0 _= *n*_0_/*n *and *f*_1 _= *n*_1_/*n*_1_/*n*,

and the *gini impurity *of this node, Φ(*η*), can be calculated as

$$\Phi (\eta )=\left(1-f_{0}^{2}-f_{1}^{2}\right)/2=f_{0}f_{1}.$$

This formula can be generalized to account for three or more classes [34]. Now, for a split at this node that yields two sub-nodes *η*_*l *_and *η*_*r*_, the decrease of the gini impurity for this split is calculated as

ΔΦ(*η*) = Φ(*η*) - *f*_*l *_Φ(*η*_*l*_) - *f*_*r *_Φ(*η*_*r*_),

where *f*_*l *_and *f*_*r *_are the fractions of samples in *η *that fall into *η*_*l *_and *η*_*r*_, respectively. Since the split is happen on a certain feature *v*, this decrease in gini impurity is also defined as the *gini decrease *for *v *at the node *η*. Moreover, *v *may be used as the splitting variable in more than one node. Let I (*η*, *v*) be the indicator function that is equal to 1 when *v *is the splitting variable of *η *and 0 otherwise. The gini decrease of *v *in this tree is then defined as the summation of gini decreases for all nodes in which *v *is the splitting variable, as

$$\text{GD}(T,v)=\sum_{\eta \in N_{T}}\Delta \Phi (\eta )\text{I}(\eta ,v),$$

where **N**_*T *_is the collection of all nodes of the tree *T*. Finally, the summation of all gini decrease of *v *over all trees in the forest is the gini importance of *v*, as

$$\text{GI}(v)=\sum_{T\in T}\sum_{\eta \in N_{T}}\Delta \Phi (\eta )\text{I}(\eta ,v)$$

where **T **is the collection of all decision trees in the random forest.

The random forest provides another randomization mechanism to estimate the importance of individual features. When a decision tree is constructed, the correct classifications for the OOB samples can be counted. Now, for a feature *v*, randomly permute its values in the OOB samples and again count the correct classifications. The average of the difference in these two counts over all trees in a forest is then defined as the *raw importance *of the feature *v*.

It has been shown that the gini importance and the raw importance are very consistent [28], but the computation of the gini importance is much more economy. We therefore adopt the gini importance to measure the contribution of a SNP to the classification performance in our studies.

### Sliding window sequential forward feature selection

The key step in *epi*Forest is to automatically determine a subset of candidate SNPs that are likely to be linked to the disease. We use a sliding window sequential forward feature selection (SWSFS) algorithm on the basis of the gini importance for this purpose.

Suppose that **M **= {*m*_1_,..., *m*_*L*_}. are a number of *L *markers, and through an initial run of a random forest, the gini importance for these markers has been obtained as **G **= {*g*_1_,..., *g*_*L*_} , where *g*_*i *_= GI(*m*_*i*_). Besides, the order of these markers on the basis of the importance is obtained as **O **= {*o*_1_,..., *o*_*L*_}, that is, for every (*i*, *j*) that satisfies 1 ≤ *i *<*j *≤ *L*, $g_{o_{i}}\geq g_{o_{j}}$. In other words, $m_{o_{1}}$ is the most important marker, $m_{o_{2}}$ the second most important one, and so forth. A *Naïve Greedy Sequential Forward feature Selection *(NGSFS) algorithm can then be designed as follows:

Naïve Greedy SFS

1.    *i *:= 1; *k *:= 1;

2.    WHILE (*i *≤ *L*) DO

3.       Error [*i*] := randomForest(**M **[*o*_1_,..., *o*_*i*_]);

4.       IF (Error [*i*] < Error [*k*])

5.          *k *:= *i*;

6.       END IF

7.    END WHILE

8.    RETURN **M **[*o*_1_,..., *o*_*k*_];

Here, **M **[*o*_1_,..., *o*_*i*_] is the subset of the first *i *most important SNPs, **M **[*o*_1_,..., *o*_*k*_] the subset of selected SNPs, and *k *the number of selected SNPs.

This algorithm greedily searches for a subset of SNPs that can minimize the classification error instead of enumerating all 2^*L *^- 1 nonempty subsets of the *L *SNPs. However, when *L *is huge, even this algorithm is computationally intractable. In our studies, we find that the classification errors produced by the NGSFS have a "V" shape with many local minimum, and the true global minimum is typically produced when only a small number of the most important SNPs are used (see Figure 6 as an example). Also, the construction of a random forest with fewer features is faster. These observations motivate us to propose the following *Sliding Window Sequential Forward feature Selection *(SWSFS) algorithm:

Sliding Window SFS

1.    *i *=: 1; *k *=: *L*; *w *=: 20;

2.    WHILE (*i *≤ *L*) DO

3.       Error [*i*] =: randomForest(**M **[*o*_1_,..., *o*_*i*_]);

4.       IF (*i *> *w *AND *i *- *w *= argmin_*i*-*w *≤ *j *≤ *i *_{Error [*j*]})

5.          *k *=: *i *- *w*;

6          BREAK;

7.       END IF

8.    END WHILE

9.    RETURN **M **[*o*_1_,..., *o*_*k*_];

This algorithm greedily searches for the first subset of markers in which the left boundary should have the minimal classification error in a window of size *w*. The window size determines how robust the algorithm could be, and we simply set it to 20 in this paper.

### Statistical tests

We adopt a hierarchical procedure on the basis of the *B *statistic [17] in the second stage of *epi*Forest to declare statistical significance of up to three-way interactions within the candidate SNPs that are selected by the SWSFS algorithm.

There are two motivations for using the *B *statistic: (1) it is more powerful than the standard *χ*^2 ^statistic, and (2) the marginal effects of already reported individual SNPs or partial interactions can be handled via the use of a conditional *B *statistic. Here we briefly introduce the *B *staistic, while the detailed derivation should refer to [17].

Given a set Ω of *k *markers, we like to test the hypothesis *H*_0_: SNPs in Ω are not associated with the disease versus *H*_1_: SNPs in Ω are jointly linked to the disease. For this purpose, a *B *statistic is defined as

*B*_Ω _= ln [*P*_1_(*D*_Ω_, *U*_Ω_)/*P*_1_(*D*_Ω_, *U*_Ω_)],

where *D*_Ω _and *U*_Ω _are the observed case and control data for the set of markers, respectively, and *P*_1_(*D*_Ω_, *U*_Ω_) and *P*_0_(*D*_Ω_, *U*_Ω_) are Bayesian factors (marginal probabilities of the data) under the alternative and the null hypotheses, respectively.

More specifically, *P*_1 _(*D*_Ω_, *U*_Ω_) is calculated as

*P*_1 _(*D*_Ω_, *U*_Ω_) = *P*_join _(*D*_Ω _[*P*_ind _(*U*_Ω_) + *P*_join _(*U*_Ω_)],

and *P*_0_(*D*_Ω_, *U*_Ω_) is calculated as

*P*_0 _(*D*_Ω_, *U*_Ω_) = *P*_ind_(*D*_Ω_, *U*_Ω_) + *P*_join_(*D*_Ω_, *U*_Ω_).

Here,

$$P_{\text{ind}}(U_{\Omega })=\prod_{i=1}^{k}\left(\frac{\Gamma \left(\sum_{j=1}^{3}\alpha_{j}\right)}{\Gamma \left(N_{u}+\sum_{j=1}^{3}\alpha_{j}\right)}\prod_{j=1}^{3}\frac{\Gamma \left(n_{ij}+\alpha_{j}\right)}{\Gamma \left(\alpha_{j}\right)}\right),$$

and

$$P_{\text{join}}(U_{\Omega })=\frac{\Gamma \left(\sum_{j=1}^{3^{k}}\beta_{j}\right)}{\Gamma \left(N_{u}+\sum_{j=1}^{3^{k}}\beta_{j}\right)}\prod_{j=1}^{3^{l}}\frac{\Gamma \left(n_{j}+\beta_{j}\right)}{\Gamma \left(\beta_{j}\right)}.$$

In the above formulae, *α*_*j *_and *β*_*j *_are pseudo-counts with default values 0.5 (see [17]), *n*_*ij *_the number of controls that have the *j*-th genotype at the *i*-th SNP, and *n*_*j *_the number of controls that have the *j*-th combinatory genotype. *P*_join_(*D*_Ω_), *P*_ind_(*D*_Ω_, *U*_Ω_), and *P*_join_(*D*_Ω_, *U*_Ω_) can be calculated in a similar way. These formulae are derivation from a Bayesian marker partition model in [17]. It has been shown that under the null hypothesis, 2*B*_Ω _has asymptotically a shifted *χ*^2 ^distribution with 3^*k *^- 1 degrees of freedom. A *p*-value can therefore be calculated using the *χ*^2 ^distribution.

Give a subset *ω *of Ω, where the *t *markers in *ω *are linked to the disease through either individual and/or partial interactive effects. A conditional *B *statistic, *B*_Ω|*ω*_, for the rest markers can then be calculated in a similar way as the *B *statistic. Furthermore, under the null hypothesis that the rest markers are unlinked to the disease, 2*B*_Ω|*ω *_follows asymptotically a shifted *χ*^2 ^distribution with 3^*k *^-3^*t *^degrees of freedom. A *p*-value can then be calculated accordingly.

## Competing interests

The authors declare that they have no competing interests.

## Authors' contributions

RJ designed the research, performed the simulation studies and the real application to AMD, and prepared the manuscript. WT implemented the disease models. XW participated in the research design and helped to prepare the manuscript. WF performed preliminary studies for the application to AMD. All authors read and approved the manuscript.
